# A Review of Most Relevant Complications of Transcatheter Aortic Valve Implantation

**DOI:** 10.1155/2013/956252

**Published:** 2013-05-12

**Authors:** Siyamek Neragi-Miandoab, Robert E. Michler

**Affiliations:** Department of Cardiovascular and Thoracic Surgery, Montefiore Medical Center, Albert Einstein College of Medicine, 3400 Bainbridge Avenue, MAP 5, New York, NY 10467, USA

## Abstract

Transcatheter aortic valve implantation (TAVI) has emerged for treating aortic stenosis in patients who are poor candidates for surgical aortic valve replacement. Currently, the balloon-expandable Edwards Sapien valve—which is usually implanted via a transfemoral or transapical approach—and the self-expanding CoreValve ReValving system—which is designed for retrograde application—are the most widely implanted valves worldwide. Although a promising approach for high-risk patients, the indication may be expanded to intermediate- and eventually low-risk patients in the future; however, doing so will require a better understanding of potential complications, risk factors for these complications, and strategies to individualize each patient to a different access route and a specific valve. This paper reviews the most relevant complications that may occur in patients who undergo catheter-based aortic valve implantation.

## 1. Introduction

Although surgical aortic valve replacement (SAVR) carries low morbidity and mortality rates, some patients are not surgical candidates and/or carry a high risk [1–3]. With the advent of transcatheter aortic valve implantation (TAVI), many high-risk patients have become eligible for AVR [4]. The early results of landmark studies demonstrated that TAVI improves hemodynamics and is an alternative to SAVR in high-risk patients [5–8]. Many patient characteristics (as seen in most cited series) are presented in Tables 1, 2, and 3 [5, 9–19]. 

Coronary artery disease mandates revascularization at the time of AVR. The indication for TAVI has expanded to patients who have had previous cardiac surgery [20, 21]. PCI before TAVI can be performed as staged or simultaneously with no increased mortality [22, 23]. In a series of 125 patients who underwent TAVI with CoreValve (PCI + TAVI; *n* = 55 versus TAVI only; *n* = 70), the 30-day mortality was 6% for patients who had TAVI only versus 2% for patients treated with PCI + TAVI [22]. 

Risk-scoring systems have been utilized to create some algorithms to select very-high-risk patients who would be appropriate candidates for TAVI. The logistic EuroSCORE (LES) and the Society of Thoracic Surgeons Predicted Risk of Mortality (STS-PROM) are the standard scoring systems. Some other risks include liver disease, frailty, porcelain aorta, and previous radiation; these have not yet been properly addressed in current scoring systems [24]. Considering the recent developments in this field, a new scoring system may be necessary to identify the best candidates for TAVI with a potential to expand the indication to intermediate-risk patients and possibly lower risk patients. Preoperative morbidities of patients who underwent TAVI using Edwards SAPIEN and CoreValve in the most relevant series in the current literature are shown in Tables 4 and 5 [5, 9–19].

## 2. Most Widely Used Valves

The two most widely used valves for TAVI are the Edwards Sapien valve (Edwards Lifesciences, Irvine, CA, USA) and the CoreValve (ReValving Technology Medtronic Inc., Minneapolis, MN, USA) [4, 25, 26]. The Sapien valve can be used for antegrade (transapical) or for retrograde (transfemoral, transsubclavian, or transaortic) approaches. It has been implanted in the pulmonary artery or inside a degenerative biologic prosthesis in aortic as well as in mitral position recently [4, 27, 28]. Many devices with a self-expanding frame have been introduced into clinical practice [4, 29, 30]. Of these, the CoreValve for retrograde implantation has been the most widely used [31]. The CoreValve is a self-expanding device whereas the Edwards Sapien valve requires a balloon to expand it [32]. Early experience with larger delivery sheaths (>18 Fr) demonstrated a relatively high incidence of vascular complications with a negative impact on survival. The new generation of delivery devices has smaller diameter in order to reduce vascular complications. The CoreValve products have a broad range of valve sizes to fit to annular diameters of 18 mm to 31 mm and can be implanted via an 18 Fr catheter [33, 34]. 

## 3. Delivery Route

**Figure 1:**
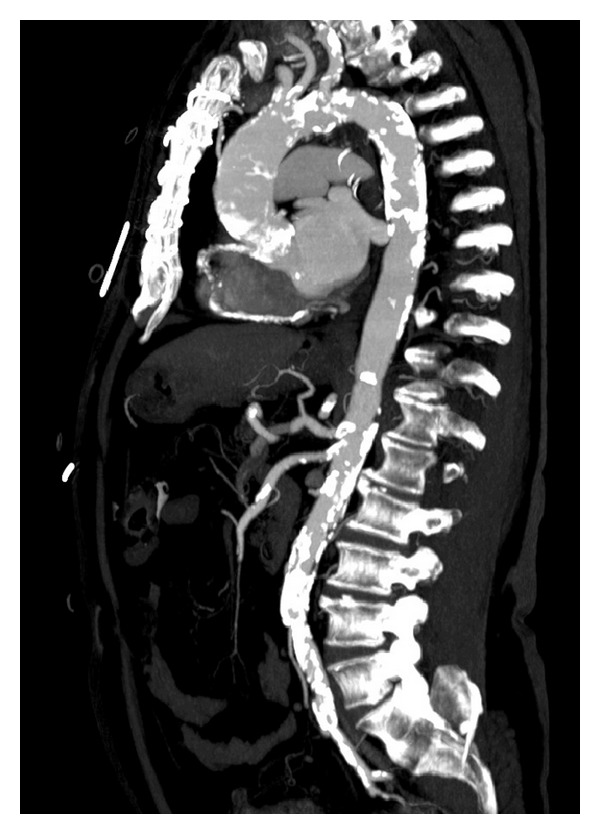
Severe calcifications of femoral and iliac vessels as well as aorta, aortic arch, and annulus, which carry a high risk of vascular complications, embolic stroke, and paravalvular leak. A transapical approach would be safer in this scenario.

One of the unique aspects for successful TAVI involves the ability to secure an access route for deployment of the aortic valve. TAVI can be performed through many access routes including retrograde transfemoral, retrograde transsubclavian, retrograde transaortic, and antegrade transapical [11, 26, 35, 36]. The transfemoral approach has been the most widely used and is commonly the first choice for access. Because of the large delivery system, it is crucial to carefully evaluate the iliofemoral vessels as well as the amount of atherosclerosis and plaque in the aortic arch and ascending aorta [37]. In cases of severe calcifications of femoral and iliac arteries as well as the aorta (Figure 1), the transapical (TA-) AVI is a viable alternative [38]. The TA-AVI reduces the vascular complications; further, shorter catheter length and using the antegrade approach may allow for more precise control of the device [39]. A transsubclavian approach for TAVI is a reasonable option in patients with peripheral vasculopathy [40, 41]. Petronio et al. [41] compared the outcome of TAVI with the subclavian approach (*n* = 141) with a propensity matched group of 141 patients who had undergone TAVI with the transfemoral approach. The two groups showed similar procedural success; however, incidence of acute kidney injury (AKI) stage III, vascular complications, and bleeding was lower with the subclavian approach. The midterm survival rate and freedom from cardiovascular death were similar in both groups.

## 4. Complications

The recently introduced Valve Academic Research Consortium (VARC) 1 [44] and 2 [45] criteria may help to standardize documentation of postoperative complications like myocardial infarction, stroke, bleeding, acute kidney injury, vascular complications, and valve performance, as well as the risk of mortality [44, 45]. Malpositioning, valve migration/embolization, conversion to open surgery, renal failure, need for pacemaker implantation, stroke, and myocardial infarct are other major complications following TAVI [27]. Blocking the coronary ostia, limiting the anterior mitral leaflet mobility, and atrioventricular conduction system are some frequently encountered perioperative complications [39, 46].

**Figure 2:**
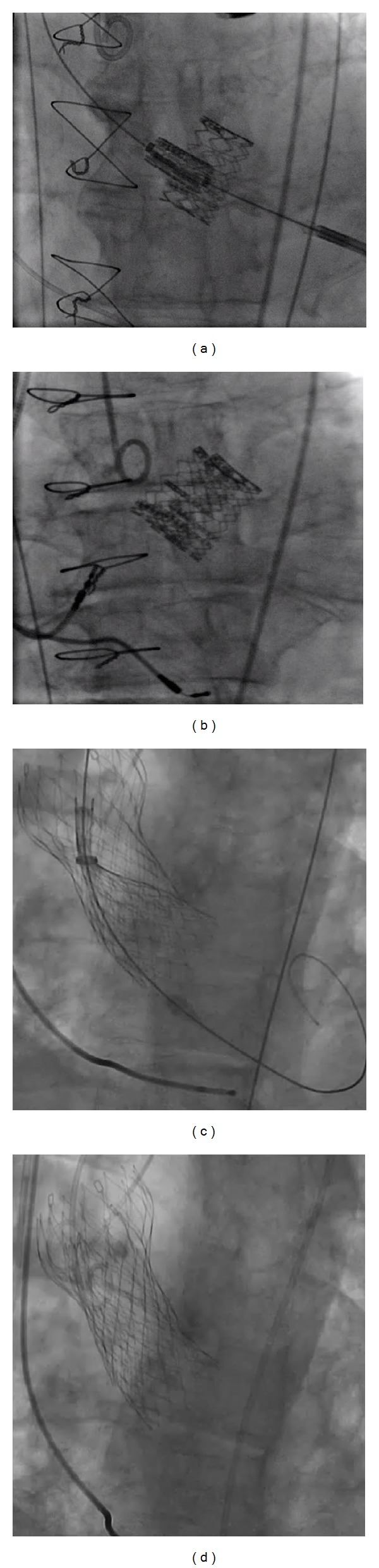
Valve-in-valve bailout procedure. (a) and (b) showing the valve-in-valve for Sapien valve and (c) and (d) for CoreValve.

**Figure 3:**
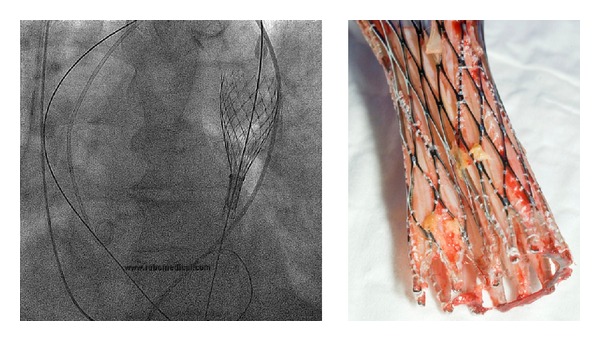
Valve retrieval. A retrieval of CoreValve is possible if the valve is not completely released; however, this maneuver carries a high risk for vascular complications and embolic stroke.

Prosthesis dislocation during TAVI is a rare but serious complication. It can be managed effectively by implanting a second device (Figure 2) and leaving the dislocated device safely in the aorta or complete retrieval of valve (Figure 3) [47]. In series of 181 patients (the Italian CoreValve Registry) it was shown that patients who experienced major or life-threatening bleeding after procedure had a higher rate of mortality. Patients with renal insufficiency, defined by VARC 1 [44] and VARC 2 [45] criteria, had a higher mortality rate at 3-year followup (49% versus 29%) [48]. Patients with LES > 20 also have higher mortality (25.7% versus 6.8%) at 12 months compared with patients with LES < 20 [43]. In addition to LES, renal disease, liver disease, low baseline LVEF, and smoking are risk factors for 1-year mortality [12, 49–51]. Tables 5 and 6 demonstrate the postoperative outcomes and most frequent complications in some of the major series in the current literature. 

### 4.1. Vascular Complications

**Figure 4:**
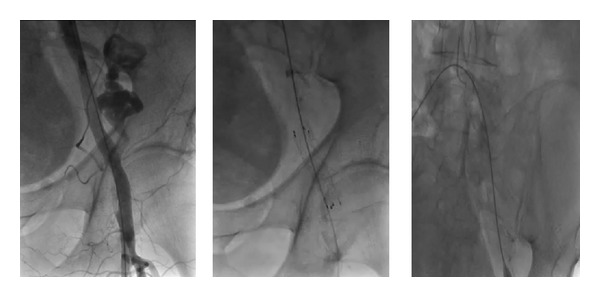
Perforation of left femoral artery and stent placement. A stent placement is easier and faster in presence of a crossover wire in the femoral artery.

**Figure 5:**
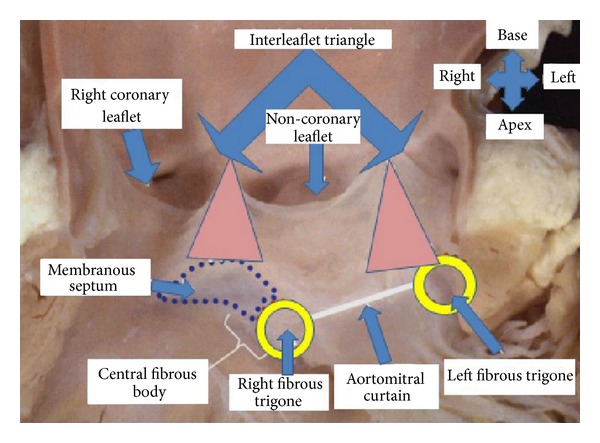
The anatomy of aortic valve, aortic root, coronary arteries orifice, and the conduction system. The proximity of coronary orifice and conduction system to the annulus may explain some of the complications of TAVI.

The transfemoral approach is associated with higher vascular complications compared to the TA-TAVI (Figure 4), [5, 39, 50, 52–54] although the trend toward reduced sheath size has shown a significant reduction in vascular complications [55]. Proper patient selection by using appropriate preoperative imaging (vascular CT scan and angiography) may reduce vascular complications [55]. Considering the fragile condition of patients who undergo TAVI, a percutaneous arterial closure device (Prostar) has been used to limit burden of the procedure; [55, 56] however, this approach has been reported to be associated with increased vascular complications [52]. In a series of transfemoral-TAVI, the percutaneous approach using Prostar was performed in 142 patients who underwent TAVI with Sapien valve (*n* = 109, sheath size 18–24 F) or CoreValve (*n* = 31, sheath size 18 F); vascular complications occurred in 20% of participants, 3.6% of whom required surgical repair. A transfemoral approach with Sapien valve carries a higher risk for Prostar failure and vascular complications [52]. Other risk factors for life-threatening bleedings following TAVI include female gender, using a larger size delivery system (>19 Fr), peripheral arterial disease (PVD), valve retrieval (Figure 3), and percutaneous access [56]. The size of the delivery system has been reduced in recent years from previously 24 French to the actual 18-19 F [55]. Even smaller introducers have been announced by manufacturers and are anticipated to be available in near future. 

Vascular complications after TAVI can be treated percutaneously with high technical success and acceptable clinical outcomes (Figure 4). In a series of 149 TAVI patients, the transfemoral percutaneous approach was associated with vascular complications in 27 patients (18%). After a median followup of 10.9 months, imaging studies showed no evidence of hemodynamically significant stenosis in repaired femoral vessels [57]. Bleeding after TAVI is mostly related to vascular complications [53, 58]. Blood transfusion following TAVI is associated with increased mortality at 1 year and increased risk of major stroke and acute kidney injury [59]. Reduced bleeding and less need for blood transfusion may improve outcomes in TAVI patients. Specific scores are needed to identify the patients who are at higher risk for TAVI-related vascular complications; this knowledge would enable providers to select a different access route in these patients when appropriate. 

### 4.2. Stroke

Stroke remains a troublesome adverse event following TAVI. It is more frequent among patients who undergo TAVI than SAVR [5, 50, 54, 60] and is associated with reduced survival [61]. Cerebrovascular accidents occur mostly during the procedure or shortly thereafter and are more frequent with repeated attempts to implant the prosthesis [61]. TAVI causes a substantial amount of cerebral microemboli; importantly, the high number of the microemboli may correlate with the severity of the postprocedural cerebral injury [62, 63]. In a series of 389 patients, Pilgrim [64] reported that age > 80 years, body mass index > 20 kg/m^2^, prior stroke, and atrial fibrillation (AF) may increase the risk of CVA in patients undergoing TAVI [64]. Stortecky et al. [61] reported a stroke incidence of 3.6% in a series of 389 patients. Patients with CVA had an increased risk of all-cause (42.3% versus 5.1%) and cardiovascular mortality (38.4% versus 4.6%) compared to patients without CVA at 30-day followup [61]. In a series of 214 patients who underwent TAVI using the CoreValve, stroke occurred in 19 patients (9%) in the perioperative period. New-onset AF and baseline aortic regurgitation grade III or greater increased the risk of stroke [65]. In a larger series of 1,061 patients, cerebrovascular events (CVE) occurred in 54 patients (5%) within 30 days of TAVI. The predictors of CVE in the acute/subacute period were postdilation of the prosthesis, valve embolization, and new-onset AF. Late CVEs occurred in 35 patients (3.3%) at a median followup of 12 (3–23) months. The predictors of late CVEs were chronic AF, PVD, and prior cerebrovascular disease (CVD). In a meta-analysis including 53 studies with a total of 10,037 patients who had undergone transfemoral, transapical, or transsubclavian TAVI, Eggebrecht et al. [40] reported that TAVI was associated with an average 30-day CVA of 3.3%. The incidence of stroke was associated with access route; the lowest stroke rate was observed with the transapical approach (2.7%). A major stroke following TAVI is associated with increased mortality within the first 30 days [40, 61, 66].

Evaluation of cerebral microembolism following TAVI with magnetic resonance imaging (MRI) has demonstrated new foci of reduced diffusion. Reinsfelt et al. [63] reported that 37% of the instances of microembolism occurred during manipulation of the aortic arch/root/valve by guide wires and catheters, 22% occurred after balloon dilatation of the valve, and 41% occurred during implantation of the prosthesis. However, despite the evidence of microemboli, none of the patients developed neurological symptoms [63]. Multiple studies have shown no correlation between MRI-detected embolic events and a clinical CVA [60, 67, 68]. Recently, diffusion-weighted MRI (DW-MRI) studies have confirmed the phenomenon of new perfusion deficits and microemboli after TAVI [67, 69]. Following TAVI, many patients have demonstrated a higher rate of cognitive decline compared to SAVR; however, the brain lesions per patient and cumulative embolic load per patient in DW-MRI were not associated with postoperative cerebral microischemia, cognitive dysfunction [60], or increased mortality [67]. In a series of 39 patients [67], the DW-MRI following TAVI showed new embolic events in 72.0%; however, only 6.6% of those patients had any clinically significant neurological deficits [67]. These “silent” cerebral infarctions occur frequently after TAVI yet have no clinical relevance [67, 68]. In another series of 31 TAVI patients evaluated with MRI, multiple small (silent) cerebral infarcts occurred in 77% of patients. Patients who had multiple large embolic events were shown to have suffered a clinical stroke. Advanced age and severity of calcifications on the valve and aortic arch may increase the risk of embolic events following TAVI [68].

A protection device that can be placed on the aortic arch (inserted through radial (7 F) or contralateral femoral artery (9 F)) may reduce the incidence of embolic events during TAVI [62]. The vast majority of embolic events and strokes are caused by embolization of atherosclerotic material and other debris from the stenotic valve during various phases of TAVI. Recently, Onsea et al. [70] reported their experience with a protection device (SMT Embolic Deflection Device) in 15 TAVI patients. A brain diffusion weighted (DW)-MRI detected 3.2 new cerebral lesions per patient who had an SMT filter placed compared to 7.2 new lesions per patient in the group without an SMT filter. None of their patients, both with and without SMT filter, developed new onset permanent neurological deficits or clinical findings of stroke; only 1 patient suffered a transient ischemic attack (TIA) [70]. Naber et al. [62] reported on the safety of the Claret CE Procerebral protection device (Claret Medical, Inc., Santa Rosa, CA, USA) in 35 patients undergoing TAVI. Evidence of captured debris was documented in at least 19 of 35 implanted devices (54.3%). No periprocedural TIA, minor strokes, or major strokes were reported. Thirty-day followup showed that one minor stroke had occurred early in the 30 days after the procedure, and two major strokes occurred later in the 30 days after the procedure [62]. Considering the high cost of the cerebral protection devices, the expertise required to implant it, and the lack of documented benefits of using it, selecting the device should be individualized to each patient's condition, amount of calcification on the valve, and a history of previous cerebrovascular disease.

### 4.3. Renal Failure

TAVI has been shown to increase the risk of AKI (acute kidney injury) defined by the VARC 1 [44] and 2 [45] criteria. The incidence of AKI following TAVI ranges from 12% to 21% in different series and is associated with increased 30-day and 1-year mortality [13, 71–73]. Although in the majority of the cases AKI is reversible, AKI stage III may worsen the 1-year survival [72]. The predictors of AKI in TAVI patients include a history of diabetes mellitus, PVD, and advanced renal insufficiency [71]. In a prospective study of 150 TAVI patients (using CoreValve), Nuis et al. [49] reported a 30-day AKI of 19%. Some authors reported a higher incidence of AKI following transapical TAVI [72]. Blood transfusion increased the risk of AKI following TAVI, which indicated that the outcome of TAVI may be improved by more restrictive use of blood transfusions [73, 74]. In a multicenter study evaluating 995 TAVI patients (CoreValve and Sapien valve), AKI occurred in 20.7% (*n* = 206) [73]. The number of units of blood transfusion was the strongest predictor of AKI; the second and third strongest predictors were PVD and a history of heart failure, respectively. AKI and life-threatening bleeding were independent predictors of 30-day mortality whereas transfusion, baseline anemia, and AKI predicted mortality beyond 30 days [73]. In their series of 102 patients of whom 87.3% had chronic kidney disease, Saia et al. [72] reported that a periprocedural AKI developed in 42 patients: 66.7% in the transapical group, 30.3% in the transfemoral group, and 50.0% in the transsubclavian group. The transapical approach was a significant predictor of AKI, but the strongest predictor of 1-year mortality was the postprocedural AKI III grade [72]. Other authors reported the transapical approach to be a risk factor for AKI [64, 74]. However, these statements might be biased because all patients who undergo the transapical approach have an advanced PVD that precludes the transfemoral approach. Thus, this association may only reaffirm the general condition of patients and the calcifications of certain arteries including renal arteries. The PVD and the number of units for blood transfusion have been reported to be risk factors for AKI after TAVI [71, 73]. Further, a transapical approach may require more blood transfusion due to the nature of the procedure. 

TAVI does not seem to increase the risk of morbidity and mortality in patients with end-stage renal disease (ESRD); therefore, it should be considered as an alternative to open surgery in dialysis patients [75]. Wessely et al. [75] evaluated the outcome of TAVI in patients with ESRD; all dialysis patients survived [75]. However, the patients with ESRD should be differentiated from patients with renal insufficiency, who still depend on their remaining kidney function; moreover, an AKI in the setting of a chronic kidney disease needs meticulous management.

### 4.4. Paravalvular Leak

The irregular surfaces of a native, calcified aortic valve may prevent a sealing between the prosthesis and the annulus, thereby increasing the risk of paravalvular leak following TAVI. During TAVI, the native valve is crushed against the aortic wall and into the sinuses of valsalva. These debris and calcifications are eventually trapped between the annulus and the prosthesis. Thus, a slight prosthesis insufficiency is not uncommon (i.e., reported in about 70% of patients for all types of valves used for TAVI) [16]. Annular calcification and bicuspid aortic valve are significant risk factors for paravalvular leak following TAVI [76, 77]. Thus, preoperative cardiac CT is needed to assess the degree of calcification, which may predict the patient's risk of paravalvular leak. An accurate measurement of the aortic annulus is also crucial for reducing the risk of prosthesis mismatch (Figure 5, anatomy of the aortic root) [78]. An underexpansion of the prosthesis stent frame is a major risk factor for paravalvular leak, which might be caused by calcifications of the annulus or of the cusps of the native valve, prosthesis malposition (implantation depth, that is, too deep or too shallow), and/or annulus-prosthesis-size mismatch. Recently published studies report an incidence of moderate/severe paravalvular leak of 15% to 20% following TAVI [5, 16, 79–82]. Evaluation of the severity of a paravalvular leak following valve implantation is critical; it has been shown to correlate with short- and long-term outcomes [16, 82]. In a large series of 663 patients who underwent TAVI with CoreValve, a postimplantation paravalvular leak of 2 or greater was a major risk factor for 30-day and 1-year mortality [16]. Using a larger-diameter prosthesis to overstretch the aortic annulus into a rounder shape may improve the attachment between the prosthesis and the annulus and may reduce the risk of paravalvular leak; yet this maneuver would increase the risk of embolizing the debris from the calcified valve into the coronary arteries or rupture the aortic annulus. With a severe paravalvular leak, assuming that the prosthesis has been correctly positioned, a post-dilatation may be necessary [76] and has been shown to improve paravalvular leaks. However, the post-dilatation has also been associated with a minimal but serious risk of rupture of the aortic annulus. 

Recently, some authors have reported the efficacy of the aortic regurgitation (AR) index to estimate the significance of paravalvular leak and its impact on 1-year mortality after TAVI. The AR index can provide additional prognostic information to complement the echocardiographic assessment of paravalvular leak [83]. Sinning et al. [83] studied paravalvular leak in 146 TAVI patients with CoreValve. In addition to echocardiographic evaluation of paravalvular leak, the AR index was calculated as a ratio of the gradient between diastolic blood pressure (DBP) and left ventricular end-diastolic pressure (LVEDP) to systolic blood pressure (SBP): [(DBP – LVEDP)/SBP] × 100. An AR index <25 had a significantly increased 1-year mortality risk compared with an AR index ≥25 (46.0% versus 16.7%) [83]. Echocardiographic paravalvular leak remained a significant predictor of 1-year mortality; [83] a combination of echocardiographic paravalvular leak and AR index predicted the risk of mortality more precisely. Patients with moderate/severe paravalvular leak and an AR index <25 had the worst outcomes, with a 1-year mortality rate of 70% [83]. It should be noted that AR index depends on LVEDP and other hemodynamic parameters, which might be affected by preexisting cardiac conditions. 

In addition to calcifications of the annulus and the native valve, prosthesis malposition, and/or annulus-prosthesis-size mismatch are some of the risk factors for paravalvular leak [79, 82, 84]. 

Some authors have recommended a certain degree of prosthesis oversizing [79, 82, 84] for an adequate adaptation of the prosthesis to the aortic annulus. Conversely, recent data have shown that oversizing of the valve was not able to reduce the incidence or the severity of paravalvular leak [81], which indicates that the paravalvular leak is a multifactorial issue and mandates that surgeons consider each case individually in terms of size, type of valve, and access. Correction of a deep implantation depth of the prosthesis can be overcome by using snare catheters or repositioning the prosthesis; by contrast, the implantation of a second prosthesis (“valve-in-valve”) should only be considered as a last resort for misplaced or embolized valves [79]. 

### 4.5. AV Block

An atrioventricular (AV) block requiring a permanent pacemaker (PPM) implantation occurs in 10–50% of patients following TAVI [85, 86]. Post-TAVI electrocardiogram monitoring should be continued for a few days, especially in patients with higher risk of AV block [48]. This conduction disturbance is caused by damage to the bundle of His or the AV node [87]. Figure 5 shows the anatomic relationship of the aortic annulus and its proximity to the conduction system. Implantation of a larger prosthesis into a smaller annulus carries a higher risk for AV block [48]. In a series of 151 patients who underwent TAVI using a Sapien valve (either transapical or transfemoral), the incidence of complete AV block was 5.3% [50]. The AV-block incidence and need for PPM implantation are higher with CoreValve implantation [88–90]. This observation was confirmed by Erkapic et al. [91] in a meta-analysis including 5,258 patients from 32 studies (Sapien valve *n* = 2,887 and CoreValve *n* = 2,371). The incidence of PPM implantation after TAVI was 15.0%, 25.8% after CoreValve, and 6.5% after Sapien valve implantation [91]. A preexisting right bundle branch block (RBBB) is associated with higher risk of postprocedural AV block and subsequent pacemaker implantation [32, 89, 90, 92, 93]. In addition to RBBB and type of valve, a deep valve implantation (<6 mm from the lower edge of the noncoronary cusp to the ventricular end of the prosthesis) is another risk factors for periprocedural AV block [90]. The risk of postoperative AV block increases by 2-fold following a large valve implantation in a small annulus, 4-fold with using CoreValve versus the Sapien valve, and 5-fold in presence of AV block episode during the procedure [48]. Some other risk factors for significant AV block and PPM implantation following TAVI include patients' age >75 years, oversizing >4 mm, and bradycardia (<55 beats per minute) preoperatively and on the first postoperative day [85]. Calcification load of the native valve and device landing zone are other risk factors for post-TAVI AV block requiring PPM implantation [76]. The majority of AV blocks occur within 3–7 days, which underscores the importance of close electrocardiographic followup during this period [48, 91]. Almost half of AV blocks during TAVI occur with balloon dilatation; about half of these improve on first postoperative day [94]. 

## 5. Summary

Current evidence has demonstrated that TAVI is a feasible alternative to surgical aortic valve replacement in certain patients. Knowing the potential complications may help surgeons make the right decision for each patient depending on the patient's preexisting morbidities. Future clinical studies need to focus on individualizing each specific valve and access route to each patient's anatomy and general condition. Further, guided by clinical studies, the indication for TAVI may be expanded to intermediate- and lower-risk patients with aortic stenosis.

## Figures and Tables

**Table 1:** Preoperative patient characteristics.

Series	Type of valve	Route	*N*	Mean age	NYHA 3/4	NYHA 4	EF%
Makkar et al. [42]	Sapien		179	83.1	165 (92%)		53.9
Kodali et al. [14]	Sapien	244 transfemoral 104 transapical	348	83.6	328 (94%)		52.5
Thomas et al. [12, 13]	Sapien	463 transfemoral	463	81.7	353 (76%)	68 (15%)	
Thomas et al. [12, 13]	Sapien	575 transapical	575	80.7	446 (78%)	78 (14%)	
Himbert et al. [10]	Sapien	51 transfemoral	51	82	49 (96%)	22 (43%)	52
Himbert et al. [10]	Sapien	24 transapical	24	82	22 (92%)	9 (38%)	48
Grube et al. [9]	CoreValve		136	81.6	130 (96%)		51.5
Litzler et al. [15]	Sapien		61	81	44 (72%)		56.3
Tamburino et al. [16]	CoreValve	599 transfemoral 64 transsubclavian	663	81	434 (71.5%)		52.1
Avanzas et al. [17]	CoreValve		108	78.6	63 (58.4%)	22 (20.4%)	
Kempfert et al. [11]	Sapien	299 transapical	299	82	252 (84%)	53 (18%)	55.3
Gotzmann et al. [18]	CoreValve		145	79.1	138 (95%)	34 (23%)	55.8
Bleiziffer et al. [19]	Sapien and CoreValve		227	81	218 (96%)		

**Table 2:** Patients' preoperative risk factors.

Series	Type of Valve	h/o CVA	h/o PVD	Renal disease	STS score	EuroSCORE
Makkar et al. [42]	Sapien	48 (27%)	54 (30%)		11.2	26.4
Kodali et al. [14]	Sapien	95 (27%)	148 (43%)		11.8	29.3
Thomas et al. [12, 13]	Sapien		49 (11%)	121 (26%)		25.7
Thomas et al. [12, 13]	Sapien		161 (28%)	189 (33%)		29.1
Himbert et al. [10] (TF)	Sapien		4 (8%)	16 (31%)	15	25
Himbert et al. [10] (TA)	Sapien		7 (30%)	12 (52%)	18	28
Grube et al. [9]	CoreValve	11 (8%)	28 (21%)	38 (28%)	8.9	23.4
Litzler et al. [15]	Sapien	6 (10%)				27.5
Tamburino et al. [43]	CoreValve	48 (7%)	127 (19%)	154 (23%)		23
Avanzas et al. [17]	CoreValve					16
Kempfert et al. [11]	Sapien		142 (48%)	8 (3%)	12	31
Gotzmann et al. [18]	CoreValve					21
Bleiziffer et al. [19]	Sapien and CoreValve	26 (11%)	61 (27%)	48 (21%)	7	21

**Table 3:** Previous cardiac conditions.

Series	Type of valve	COPD	CAD	h/o MI	h/o CABG	h/o PCI
Makkar et al. [42]	Sapien	74 (41%)	121 (68%)	33 (18%)	58 (32%)	47 (26%)
Kodali et al. [14]	Sapien	151 (43%)	260 (75%)	92 (26%)	147 (42%)	116 (33%)
Thomas et al. [12, 13]	Sapien		220 (48%)	10 (2%)	81 (17%)	
Thomas et al. [12, 13]	Sapien		317 (55%)	10 (2%)	155 (27%)	
Himbert et al. [10] (TF)	Sapien	14 (27%)	25 (49%)	4 (8%)	11 (22%)	7 (20%)
Himbert et al. [10] (TA)	Sapien	6 (26%)	20 (87%)	11 (48%)	12 (52%)	4 (27%)
Grube et al. [9]	CoreValve		81 (60%)	35 (26%)	41 (30%)	
Litzler et al. [15]	Sapien			22 (36%)	21 (34%)	23 (38%)
Tamburino et al. [16]	CoreValve	141 (21%)	320 (48%)	143 (22%)	104 (16%)	189 (29%)
Avanzas et al. [17]	CoreValve		36 (33%)		9 (8%)	15 (14%)
Kempfert et al. [11]	Sapien	129 (43%)	159 (53%)	8 (3%)		
Gotzmann et al. [18]	CoreValve					
Bleiziffer et al. [19]	Sapien and CoreValve	52 (23%)	118 (52%)			

**Table 4:** Preoperative hemodynamic characteristics.

Series	Type of valve	Mean AV gradient (mm Hg)	Aortic valve area (cm^2^)	Mitral regurgitation (moderate-severe)	Permanent pacemaker	Pulm HTN
Makkar et al. [42]	Sapien	44.5	0.6	38 (21%)	35 (20%)	50 (28%)
Kodali et al. [14]	Sapien	42.7	0.7	66 (19%)	69 (20%)	125 (36%)
Thomas et al. [12, 13]	Sapien			73 (16%)		114 (25%)
Thomas et al. [12, 13]	Sapien			184 (32%)		172 (30%)
Himbert et al. [10] (TF)	Sapien	54	0.63			
Himbert et al. [10] (TA)	Sapien	48	0.65			
Grube et al. [9]	CoreValve	42	0.66		14 (10%)	14 (10%)
Litzler et al. [15]	Sapien	41	0.68		11 (18%)	
Tamburino et al. [16]	CoreValve	51.8		42 (6%)	42 (6%)	
Avanzas et al. [17]	CoreValve	55	0.63			
Kempfert et al. [11]	Sapien			3 (3%)		81 (28%)81
Gotzmann et al.[18]	CoreValve	46.6		83 (57%)		91 (63%)
Bleiziffer et al. [19]	Sapien and CoreValve	48	0.6			53 (23%)

**Table 5:** Postoperative outcome: cardiac and noncardiac related mortality.

Series	Type of valve	Valve in valve	Mortality 30 d	Mortality 1 yr	Cardiac mortality 30 d	Cardiac mortality 1 yr
Makkar et al. [42]	Sapien		9 (5%)	55 (31%)	8 (4%)	35 (20%)
Kodali et al. [14]	Sapien		12 (3%)	84 (24%)	11 (3%)	47 (14%)
Thomas et al. [12, 13]	Sapien	3 (1%)	29 (6%)	88 (19%)		45 (10%)
Thomas et al. [12, 13]	Sapien	19 (3%)	59 (10%)	156 (27%)		
Himbert et al. [10] (TF)	Sapien	1 (2%)	4 (8%)	6 (12%)	3 (6%)	2 (4%)
Himbert et al. [10] (TA)	Sapien	2 (8%)	4 (16%)	8 (32%)	2 (8%)	0
Grube et al. [9]	CoreValve	3 (2%)	17 (13%)	25 (18%)		
Litzler et al. [15]	Sapien		8 (13%)	16 (26%)		
Tamburino et al. [16]	CoreValve	24 (4%)	39 (6%)	99 (15%)		
Avanzas et al. [17]	CoreValve	1 (1%)	8 (7%)	15 (14%)	6 (6%)	
Kempfert et al. [11]	Sapien	17 (6%)	26 (9%)	78 (26%)		
Gotzmann et al. [18]	CoreValve		12 (8%)			
Bleiziffer et al. [19]	Sapien and CoreValve		26 (11%)	70 (25%)	4 (1.7%)	

**Table 6:** Postoperative cardiac-related complications.

Series	Type of valve	CVA 30 d	CVA 1 yr	30 days, new pacemaker	1 yr, new pacemaker
Makkar et al. [42]	Sapien	12 (7%)	19 (11%)	6 (3%)	8 (4%)
Kodali et al. [14]	Sapien	19 (5%)	27 (8%)	13 (4%)	19 (5%)
Thomas et al. [12, 13]	Sapien	11 (2%)	46 (4%)	31 (7%)	
Thomas et al. [12, 13]	Sapien	16 (3%)		42 (7%)	
Himbert et al. [10] (TF)	Sapien	3 (6%)		3 (6%)	
Grube et al. [9]	CoreValve	6 (4%)	6 (4%)		
Litzler et al. [15]	Sapien				
Tamburino et al. [16]	CoreValve		17 (3%)	110 (17%)	127 (19%)
Avanzas et al. [17]	CoreValve				38 (35%)
Kempfert et al. [11]	Sapien	2 (1%)		10 (3%)	
Gotzmann et al. [18]	CoreValve				
Bleiziffer et al. [19]	Sapien and CoreValve	7 (3%)			
